# Body composition as a predictor of chemotherapy-related toxicity in pancreatic cancer patients: A systematic review

**DOI:** 10.3389/fonc.2022.974116

**Published:** 2022-09-29

**Authors:** Stefania Rizzo, Isabel Scala, Angela Rodriguez Robayo, Marco Cefalì, Sara De Dosso, Stefano Cappio, Genti Xhepa, Filippo Del Grande

**Affiliations:** ^1^ Istituto di Imaging della Svizzera Italiana (IIMSI), Ente Ospedaliero Cantonale (EOC), Lugano, Switzerland; ^2^ Facoltà di Scienze biomediche, Università della Svizzera Italiana, Lugano, Switzerland; ^3^ Department of Radiology, Fundación Villavicencio, Rosario, Argentina; ^4^ Istituto Oncologico della Svizzera Italiana (IOSI), Ente Ospedaliero Cantonale (EOC), Bellinzona, Switzerland

**Keywords:** pancreatic cancer, chemotherapy, body composition, sarcopenia, toxicity

## Abstract

**Objectives:**

The objective of this systematic review was to assess associations between quantitative body composition measures extracted from imaging examinations and chemotherapy-related toxicity in pancreatic cancer patients. A secondary objective was to evaluate the different definitions of sarcopenia across included studies.

**Methods:**

This systematic review was conducted according to the PRISMA statement. A comprehensive literature search of three electronic databases was performed by two authors. For each eligible article, information was collected concerning the clinical setting; basic study; population characteristics; technical; body composition features evaluated; CA 19.9 tumor marker levels; chemotherapy drugs administered; toxicities (hematologic, nausea/vomiting, diarrhea, neuropathy, reduction of number of cycles, overall toxicity); association of body composition values with toxicities. The overall quality of the included studies was critically evaluated.

**Results:**

After the initial retrieval of 1137 articles, the systematic review included 12 articles (1/12 in the neo-adjuvant setting; 2/12 in the adjuvant setting; 3/12 in the metastatic setting; 2/12 in the unresectable setting; the other 4/12 included more than one clinical setting). The number of patients included ranged between 17 and 251; mean/median age ranged between 63 and 77 years; the percentage of sarcopenic patients ranged between 23 and 76%. The most frequent body composition parameter evaluated was skeletal muscle index (11/12). Chemotherapy regimens included gemcitabine (as monotherapy or in combination with other drugs); FOLFIRINOX and S-1. Among the trials including gemcitabine, 2/9 demonstrated an association with toxicity, whereas 7/9 did not; among those including FOLFIRINOX, one demonstrated associated toxicity whereas the others did not. Altogether, 4/12 papers demonstrated an association between the body composition values and the development of chemotherapy-related toxicities.

**Conclusions:**

There is a wide variability of results about the association of body composition and chemotherapy-related toxicity in PC patients. Furthermore, cut-off values to define sarcopenia in PC patients are not yet uniformly defined.

**Systematic Review Registration:**

https://www.crd.york.ac.uk/prospero/display_record.php?ID=CRD42022337753, identifier CRD42022337753.

## Introduction

Among the malignancies originating from the digestive system, pancreatic cancer (PC) is the second most frequent with 62,210 estimated new cases in the US in 2022, and the most lethal, with 49,830 estimated deaths (1). Complete surgical resection leads to better survival rates in PC patients. However, less than one-fifth of patients are considered resectable at the time of diagnosis (2, 3), and most patients will need to undergo chemotherapy, either in the neoadjuvant, adjuvant, or advanced setting.

In PC patients, a mix of inadequate nutritional intake, metabolic alterations due to malignancy, and malabsorption leads to a loss of muscle mass, also referred to as sarcopenia (4, 5), as well as to a change in the composition of distribution of muscle and fat in the patient’s body.

Body composition assessment may include evaluation of muscle mass by skeletal muscle area (SMA) and skeletal muscle index (SMI), as well as assessment of fat distribution by subcutaneous adipose tissue (SAT) and visceral adipose tissue (VAT). Body composition has been shown to correlate with prognosis in many cancer subtypes, including ovarian (6), lung (7), bladder (8) and pancreatic malignancies (9). Furthermore, in some cancer types, sarcopenia increases the toxicity of chemotherapy (10, 11), likely because drug dosing is largely based on the body surface area, that takes into account only the patient’s height and weight but ignores the relative quantity and distribution of muscle and fat. Consequently, sarcopenic cancer patients tend to receive a higher dose of chemotherapeutic agent for a relatively small lean muscle mass and are more prone to suffer toxicity (12, 13). Unfortunately, a higher incidence of toxicity eventually leads to a higher likelihood of treatment termination and hospitalization. Cancer patients undergo numerous imaging examinations at staging and during follow-up (14–16), and since computed tomography (CT) and magnetic resonance imaging (MRI) are currently considered gold standard methods in the evaluation of human body composition (17–19), this assessment can be added to the already available imaging examinations, without the need for additional exams.

Despite different definitions and a wide variability of cut-off values for the definition of sarcopenia, this is a common condition found in PC patients (20, 21) and the assessment of muscle and fat tissues has been increasingly used in this setting. Some studies have reported poorer response to treatment and worse survival in sarcopenic patients with PC treated with chemotherapy (22–25), but these results were not consistent with other experiences, reporting association of body composition with overall survival and prognosis, but not with chemotherapy-related toxicity (26).

Therefore, the main objective of this systematic review was to collect and examine all the available literature assessing associations between quantitative body composition measures extracted from imaging examinations and chemotherapy-related toxicity in pancreatic cancer patients. A secondary objective was to evaluate the different definitions of sarcopenia across studies.

## Methods

This systematic review was conducted according to the PRISMA-DTA (Preferred Reporting Items for Systematic Reviews and Meta-analysis for Diagnostic Test Accuracy) statement (27), which describes an evidence-based minimum set of items for reporting in systematic reviews and meta-analyses of diagnostic studies.

### Search strategy

Two authors (SR and AR) performed a comprehensive computer literature search of the electronic databases PubMed, Cochrane and Web of Science to find primary publications evaluating association between body composition and chemotherapy-related toxicities in pancreatic cancer. No beginning date limit or language restrictions were used; the literature search was last updated on May 7^th^ 2022; and the search was expanded by also screening the references of the retrieved articles for additional potentially eligible studies.

### Study selection

The search terms consisted of ((pancreatic cancer) OR (pancreas carcinoma)) AND ((sarcopenia) OR (body composition) OR (muscle) OR (fat) OR (adipose tissue)) AND ((complication) OR (complications) OR (chemotherapy-related) OR (adjuvant) OR (neo-adjuvant) OR (toxicity) OR (toxicities)). Articles in which body composition assessment was based on imaging examinations (CT or MRI) in pancreatic cancer patients were obtained in full for further evaluation. Studies were excluded if they were case reports, conference abstracts, reviews or short communications because they do not provide sufficient information to assess the methodological quality. Uncertainties were resolved in consensus.

### Data extraction

For each eligible article, information was collected concerning the clinical setting (neo-adjuvant, adjuvant, unresectable, metastatic); basic study data (authors, year of publication, country of origin, prospective or retrospective nature); population characteristics (number of patients, age, sex, sarcopenic status and cut-offs used); technical aspects (axial level for evaluation of body composition, software used for extraction); features evaluated (SMA, SMI, VAT, SAT, bone mineral density); mean/median CA 19.9 tumor marker levels (if reported); difference in sarcopenia between males and females; chemotherapy drugs administered; toxicities (hematologic, nausea/vomiting, diarrhea, neuropathy, reduction of number of cycles, overall toxicity); association of body composition values with toxicities and with older age, if any.

### Quality assessment

The overall quality of the included studies was critically evaluated based on the revised “Quality Assessment of Diagnostic Accuracy Studies” tool (QUADAS-2) (28). This tool comprises four domains (patient selection, index test, reference standard, and flow and timing) and each domain was assessed in terms of risk of bias, and a graph was constructed appropriately.

## Results

### Literature search

**Figure 1:**
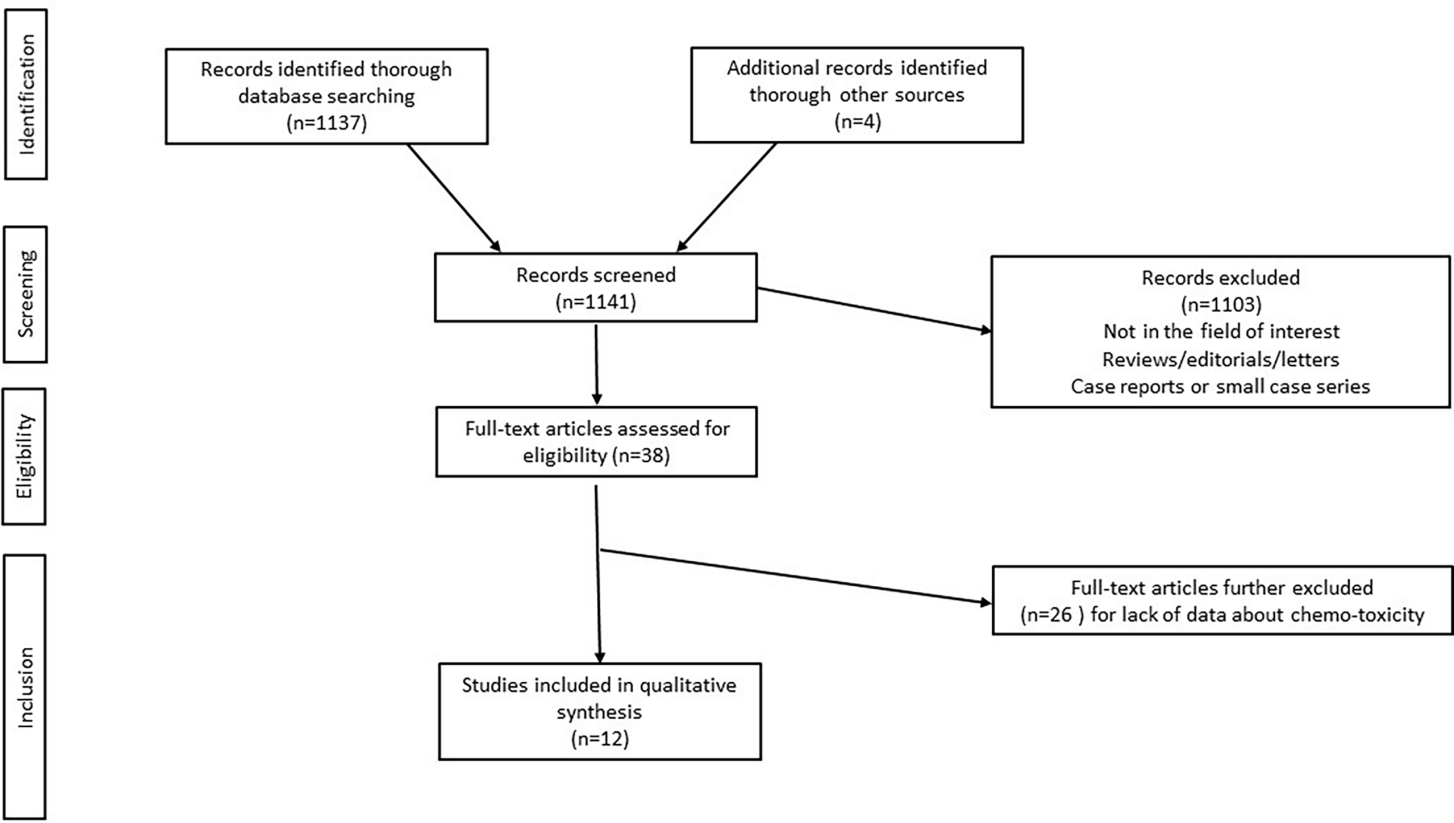
Study selection flowchart.

The initial search yielded 1137 articles, all in English. According to inclusion and exclusion criteria, 12 full-text articles were included in this systematic review (22, 26, 29–38). Details about the literature search results are reported in **Figure 1**.

Given the small number of papers included, and the heterogeneity of the quantitative analyses performed as well as of the results, a meta-analysis for pooled data was not performed.

### Clinical setting and basic study data

Among the 12 studies included, 1 was in the neo-adjuvant setting (31), 2 in the adjuvant setting (30, 37), 3 in the metastatic setting (32, 33, 36); 2 in the unresectable setting (26, 29); the other 4 included more than one clinical setting (22, 34, 35, 38). Eight of the included studies were from Japan (22, 26, 29–32, 34, 35); 1 from South Korea (33); 1 from Canada (36); 1 from Brazil (37); 1 from Australia (38); all 12 studies were retrospective (**Table 1**).

**Table 1:** Basic study and population characteristics.

Authors	Year	Country	Study design	N patients	Males/females	Mean/median age (years)	Percentage of sarcopenic patients
Asama H (26)	2022	Japan	Retrospective	124	67/57	69	51%
Emori T (29)	2022	Japan	Retrospective	176	94/82	NA	24%
Tsukagoshi M (30)	2021	Japan	Retrospective	80	43/37	72	70%
Takeda T (31)	2021	Japan	Retrospective	80	35/45	77	76%
Takeda T (32)	2021	Japan	Retrospective	62	NA	NA	40%
Kim IH (33)	2021	South Korea	Retrospective	251	161/90	63	59%
Uemura S (34)	2021	Japan	Retrospective	69	38/31	63	48%
Akahori T (35)	2015	Japan	Retrospective	83	46/37	67	NA
Youn S (36)	2021	Canada	Retrospective	152	88/64	65	63%
Barrere APN (37)	2020	Brasil	Retrospective	17	11/6	63	47%
Freckelton J (38)	2019	Australia	Retrospective	52	24/28	65	73%
Kurita Y (22)	2019	Japan	Retrospective	82	60/22	64	51%

NA, not available.

### Population characteristics

The number of patients included ranged between 17 and 251, with numbers of males and females between 11 and 161 and 6 and 90, respectively; mean/median age ranged between 63 and 77 years; the percentage of sarcopenic patients ranged between 23% and 76% (**Table 1**). The cut-off values to define sarcopenia in males and females are summarized in **Table 2**. Interestingly, the assessment of differences in sarcopenia between males and females was evaluated in 9/12 papers, and among these, 5/9 indicate a significant prevalence of sarcopenia among females, whereas the other 4/9 report no difference.

**Table 2:** Definition of sarcopenia according to skeletal muscle index in males and females within each included study.

	Males	Females
	SMI (cm^2^/m^2^)	
Asama H (26)	<42	<38
Emori T (29)	<42	<38
Tsukagoshi M (30)	<42	<38
Takeda T (31)	<43	<41
Takeda T (32)	<43	<41
Kim IH (33)	<43	<41
Uemura S (34)	<42	<38
Akahori T (35)	NA	NA
Youn S (36)	<43 if BMI<24.9 <53 if BMI >25	<41
Barrere APN (37)	<52.4	<38.5
Freckelton J (38)	<52.5	<38.5
Kurita Y (22)	<45.3	<37.1

SMI, skeletal muscle index; NA, not available; BMI, body mass index.

### Technical aspects and features evaluated

All the articles included evaluated the body composition values at the level of the third lumbar vertebra (L3); the software used was Slice-o-matic (Tomovision) in 5 studies (22, 26, 36–38) and SYN-APSE Vincent in 7 studies (29–35). As shown in **Table 3**, body composition parameters evaluated were: SMI (derived from SMA) in 11/12 studies; VAT in 6/12 studies; SAT in 7/12 studies; SMD was evaluated in 3/12 articles; bone mineral density was never evaluated.

**Table 3:** Body composition features evaluated, chemo–related toxicities encountered and their association (if any).

Authors	Body composition features evaluated	Chemotherapy	Association of body composition and chemo–related toxicity
Asama H (26)	SMA, SMI, SAT, VAT	Gemcitabine + Nab–paclitaxel	NO
Emori T (29)	SMA, SMI	Gemcitabine + Nab–paclitaxel	YES (Hematological toxicity, number of cycles)
Tsukagoshi M (30)	SMA, SMI	S–1	YES (skeletal muscle loss associated with discontinuation of chemotherapy)
Takeda T (31)	SMA, SMI	Gemcitabine, Gemcitabine + Nab–paclitaxel, S–1, FOLFIRINOX	NO
Takeda T (32)	SMA, SMI, VAT, SAT	Gemcitabine + S–1	NO
Kim IH (33)	SMA, SMI, SAT, SMD	Gemcitabine	YES (all grade 3 toxicities associated with low SMI and low SMD)
Uemura S (34)	SMA, SMI	FOLFIRINOX	NO
Akahori T (35)	SMD	Gemcitabine, 5–Fluorouracyl + Gemcitabine	NO
Youn S (36)	SMA, SMI, VAT, SAT, SMD	Gemcitabine + Nab–Paclitaxel	NO
Barrere APN (37)	SMA, SMI, VAT, SAT	Gemcitabine, Cisplatin, Oxaliplatin	NO
Freckelton J (38)	SMA, SMI, VAT, SAT	Gemcitabine + Nab–paclitaxel	NO
Kurita Y (22)	SMA, SMI, VAT, SAT	FOLFIRINOX	YES (hematological toxicity)

SMA, skeletal muscle area; SMI, skeletal muscle index; VAT, visceral adipose tissue; SAT, subcutaneous adipose tissue; SMD, skeletal muscle density.

### Chemotherapy administered, association of body composition and chemotherapy-related toxicities

Chemotherapy regimens included gemcitabine (as monotherapy or in combination with either nab-paclitaxel, cisplatin, oxaliplatin, 5-fluorouracyl or the fluoropyrimidine S-1), in 9/12 studies; FOLFIRINOX (5-fluorouracyl, irinotecan, oxaliplatin) in 3/12 studies (**Table 3**); and S-1 as monotherapy in 2/12 studies. Among the trials including gemcitabine, 2/9 demonstrated an association with toxicity, whereas 7/9 did not; among those including FOLFIRINOX, one demonstrated associated toxicity whereas the others did not. Altogether, 4/12 papers demonstrated an association between the body composition values and the development of chemotherapy-related toxicities (1 between low SMI and hematological toxicity and reduction of cycles number (29); 1 between loss of skeletal muscle and discontinuation of chemotherapy (30); 1 between low SMI and low SMD with all grade 3 toxicities (33); 1 with hematological toxicity (22)). Associations of body composition values and chemo-related toxicities are summarized in **Table 3**. Among the included articles, only 3/12 performed a specific analysis for older patients, with 2/3 study reporting no association between age and the advent of chemotherapy-related toxicity (26, 29), 1/3 reporting a significant association of chemo-related toxicity for octuagenarian patients (30).

### Quality assessment of the studies included

**Figure 2:**
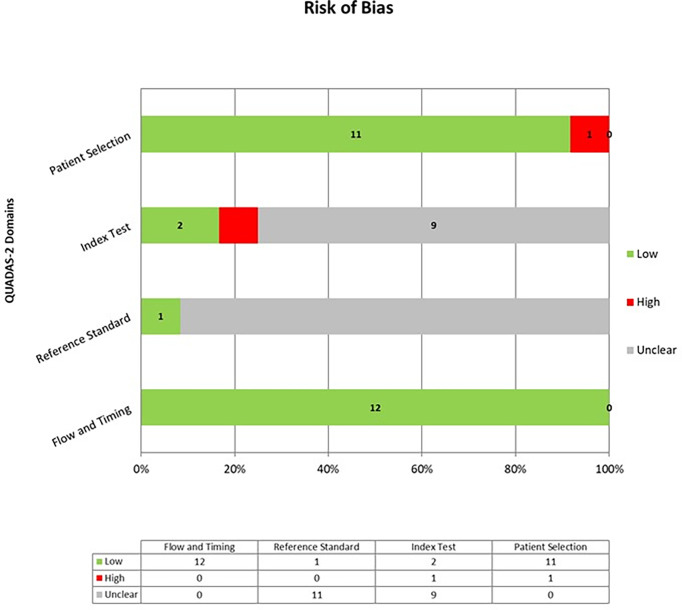
Overall Quality Assessment of the Studies Included in the Systematic Review, according to the QUADAS–2 Tool.

The overall quality assessment of the studies is reported in **Figure 2**.

## Discussion

This systematic review demonstrated that the association between body composition and chemo-related toxicity in PC is still uncertain. Indeed, 4/12 studies demonstrated the presence of a significant association, but 8/12 did not. Furthermore, Rollins et al, in a paper that we excluded because it combined patients affected by pancreatic adenocarcinoma along with patients affected by distal cholangiocarcinoma, demonstrated no significant association between sarcopenia and chemo-related toxicity in a sub-group analysis performed on 98 patients treated with chemotherapy (39).

Many different factors can explain the discrepancy of these results; for instance, the chemotherapy agent administered. Indeed, the efficacy of FOLFIRINOX and of gemcitabine/nab-paclitaxel chemotherapy has been demonstrated, but gemcitabine/nab-paclitaxel tended to cause less toxicity than FOLFIRINOX (40, 41). Accordingly, the European Society of Medical Oncology recommend FOLFIRINOX as the first adjuvant therapeutic option after resection of pancreatic cancer in selected and fit patients, in view of survival outcomes and associated toxicity profile (I, A; ESMO-Magnitude of Clinical Benefit Scale (MCBS) v1.1 score: A); gemcitabine/capecitabine as an option in less fit patients (age > 70, Eastern Cooperative Oncology Group performance status 2, or patients who have any contraindication to the drugs used in FOLFIRINOX) (I, B; ESMO-MCBS v1.1 score A); gemcitabine alone only in frail patients (42). Therefore, if gemcitabine/nab-paclitaxel is considered more appropriate than FOLFIRINOX for use as first-line chemotherapy in patients with sarcopenia and frail patients, the comparison between different populations treated with different agents may be biased.

Another possible cause of discrepancy across studies may be the segmentation method used. Indeed, the areas of muscle and fat at the level of L3 were extracted by using different software programs (SYNAPSE VINCENT or Slice-o-matic) set up at the same Hounsfield Units levels for muscle segmentation (-29 +150), but the articles did not specify whether the segmentation was semi-automatic or automatic (the semi-automatic being generally more precise but less reproducible).

Furthermore, among the studies included there is a wide variability in the proportion of sarcopenic patients. Originally, the term ‘sarcopenia’ was used to describe age-related decreases in muscle mass, but the European Working Group on Sarcopenia in Older People later defined sarcopenia as a syndrome characterized by decrease in skeletal muscle mass and strength, associated with physical disability, poor quality of life, and high mortality (4). Since the values of SMI in that definition were based on bioelectrical impedance analysis method, Martin et al, in a large cohort of lung and gastro-intestinal cancer patients (n=1473), proposed sex-specific cut-offs for lumbar SMI extracted from CT images, associated with mortality in obese and non-obese patients. Nonetheless, they included only a very small proportion of pancreatic cancer patients (9% of males and 33% of females) and therefore the values proposed may not be generalizable for all cancer patients (43). Accordingly, in 2015 the Japan Society of Hepatology decided to establish its own assessment criteria for sarcopenia in patients affected by liver disease (44). This variability in the definition of sarcopenia may cause differences in the results of different studies, and indeed this evaluation was included as a secondary objective in this study. The Asian studies referred to cut-off values defined for the Asian population, but some used slightly lower cut-off values (26, 29, 30, 34) than others (31–33). On the other hand, other studies (36–38) referred either to values elaborated in North America (43) or defined in *post-hoc* analyses (22). These discrepancies leave space for larger studies to define a proper definition for sarcopenia in PC patients, possibly according to ethnicity, in order to further assess whether an association between sarcopenia and chemo-related toxicity does exist.

This systematic review certainly has some limitations. The first is the lack of randomized trials that would clarify the role of the drug according to the sarcopenic status. However, such a study is difficult to obtain and, so far, most of the published studies, including those on other cancer types, are based on retrospective evaluations. Secondly, we did not include in this review the data about survival and general prognosis, even when available. However, since the literature search was focused on chemo-related toxicity, an evaluation of survival only in the 12 included articles would have been incomplete and even misleading, because many articles specifically dedicated to survival were excluded. Thirdly, we aimed to evaluate many body composition features, but only SMI was present in all but one (35) of the included papers, whereas data about the distribution of fat tissue was not always present or included in the statistical analysis. Nonetheless, recent advances in the extraction of body composition values such as opportunistic information from imaging studies are leading to an increasing number of dedicated studies, and we may expect more data to be published in the near future.

In conclusion, we demonstrated that there is a wide variability of results about the association of body composition and chemotherapy-related toxicity in PC patients, and more studies, hopefully prospective and including cohorts of patients treated with pre-defined agents, are warranted to better understand this association. Furthermore, uniform cut-off values to define sarcopenia in PC patients should ideally be defined, leading to more consistent results and allowing for cross-trial comparisons to a degree.

## Data availability statement

The original contributions presented in the study are included in the article. Further inquiries can be directed to the corresponding author.

## Author contributions

Conception and design: all authors. Data extraction from included studies: SR, IS, AR, MC, SC, SD. Analysis and interpretation of data: SR, MC, AR, SD. Manuscript writing: all authors. All authors contributed to the article and approved the submitted version.

## Funding

Open access funding provided by Università della Svizzera italiana.

## Acknowledgments

The English text was revised by Susan West.

## Conflict of interest

The authors declare that the research was conducted in the absence of any commercial or financial relationships that could be construed as a potential conflict of interest.

## Publisher’s note

All claims expressed in this article are solely those of the authors and do not necessarily represent those of their affiliated organizations, or those of the publisher, the editors and the reviewers. Any product that may be evaluated in this article, or claim that may be made by its manufacturer, is not guaranteed or endorsed by the publisher.
